# Repeatability of Foveal Sensitivity Measured Using Adaptive Optics Microperimetry

**DOI:** 10.1167/tvst.15.8.3

**Published:** 2026-08-05

**Authors:** Nivedhitha Govindasamy, William S. Tuten, David H. Brainard, Jessica I. W. Morgan

**Affiliations:** 1 Bioengineering Graduate Group, University of Pennsylvania, Philadelphia, PA, USA; 2 Herbert Wertheim School of Optometry and Vision Science, University of California, Berkeley, Berkeley, CA, USA; 3 Department of Psychology, University of Pennsylvania, Philadelphia, PA, USA; 4 Scheie Eye Institute, Department of Ophthalmology, University of Pennsylvania, Philadelphia, PA, USA; 5 Center for Advanced Retinal and Ocular Therapeutics, University of Pennsylvania, Philadelphia, PA, USA

**Keywords:** adaptive optics microperimetry, retinal sensitivity, photoreceptors

## Abstract

**Purpose:**

To assess the within and between-session repeatability of foveal sensitivity measurements using adaptive optics (AO) microperimetry.

**Methods:**

Five normal-sighted participants underwent AO microperimetry testing (550-nm circular stimulus) at the preferred retinal locus across two sessions (2 months ± 2 weeks). Two psychophysical paradigms were used: quick estimation of threshold (QUEST) and method of constant stimuli (MOCS). Each paradigm employed both 1.05-arcmin^2^ and 30.4-arcmin^2^ stimulus sizes; stimuli were not stabilized in real time. Trial-by-trial responses were fit with a logistic psychometric function to obtain thresholds at a criterion of 78% seen. Thresholds were converted to sensitivity. Statistical comparisons were performed using paired-sample *t*-tests.

**Results:**

Sensitivities measured from QUEST and MOCS procedures were not significantly different (QUEST: 1.05 arcmin^2^, *P* = 0.36 for session 1 and *P* = 0.41 for session 2; MOCS: 30.4 arcmin^2^, *P* = 0.14 for session 1 and *P* = 0.12 for session 2). Data were thus combined across paradigms for tests of repeatability. Sensitivities for data split into two groups within session 1 and separately within session 2 were not significantly different (QUEST: 1.05 arcmin^2^, *P* = 0.34 for session 1 and *P* = 0.51 for session 2; MOCS: 30.4 arcmin^2^, *P* = 0.31 for session 1 and *P* = 0.27 for session 2). Overall, sensitivities across the two sessions were not significantly different (QUEST: 1.05 arcmin^2^, *P* = 0.06; MOCS: 30.4 arcmin^2^, *P* = 0.81). The coefficients of repeatability across sessions were 1.5 decibels (dB) and 0.4 dB for 1.05 arcmin^2^ and 30.4 arcmin^2^, respectively.

**Conclusions:**

Sensitivity measurements from AO microperimetry were repeatable within and across sessions.

**Translational Relevance:**

Results of the present study will guide future longitudinal studies of disease progression in detecting sensitivity changes using AO microperimetry.

## Introduction

The introduction of adaptive optics (AO) technology in ophthalmology has revolutionized in vivo imaging of the human retina.^1^ Adaptive optics in combination with scanning light ophthalmoscopy (AOSLO) can achieve cellular resolution of the photoreceptor mosaic by compensating for the aberrations of the eye in real time while detecting non-confocal and confocal light reflected from the photoreceptors, thereby enabling visualization of the inner segment and waveguiding outer segment, respectively.^2^^,^^3^ In addition to providing a high-resolution view of cone mosaic structure, AOSLO technology enhanced with microperimetry allows investigators to probe cone function at a cellular scale.^4^ With AO microperimetry, diffraction-limited stimuli can be repeatedly delivered to specified retinal locations, and sensitivity can be measured for small spots confined to individual or small groups of cones.^5^

Previously, basic science psychophysical studies using the AO microperimetry platform have explored mechanisms of vision. These studies investigated the role of optical aberrations and fixational eye movements on spatial summation in the human fovea,^5^ fundamental properties of the waveguiding retina,^6^ and color vision in the trichromatic retina.^7^

AO microperimetry has also been applied to study retinal diseases including choroideremia, retinitis pigmentosa, and macular telangiectasia type 2. In choroideremia, AO microperimetry showed that visual sensitivity drops sharply in choroideremia at the atrophic border in close correlation with observed abnormalities in photoreceptor mosaic structure.^8^ On the other hand, AO microperimetry in *RPGR-*associated retinitis pigmentosa showed a structure–function dissociation with lower sensitivity despite retained cone structure.^9^ In macular telangiectasia type 2, AO microperimetry revealed retained sensitivity at retinal locations with a gap in the inner segment layer but an intact external limiting membrane as imaged by OCT. The retained sensitivity was found despite a lack of visible cones in the AO image.^10^ Common to all these studies is that the AO microperimetry technique provided knowledge about structure–function relationships at high spatial resolution in the pathological conditions examined.

To date, retinal sensitivity measured with AO microperimetry has only been reported in cross-sectional studies. Longitudinal measurements will enable structure–function correlations through the natural progression of retinal disease and its potentially altered course following a therapeutic intervention. However, before investigators can use AO microperimetry to track changes in retinal sensitivity over time in cases of retinal disease, the repeatability of AO microperimetry sensitivity measurements must be established. Thus, we designed the current study to investigate the repeatability of AO microperimetry sensitivity measurements at the fovea in normal-sighted participants. The study assessed sensitivity for two stimulus sizes delivered using two psychophysical testing paradigms—quick estimation of threshold^11^ (QUEST) and the method of constant stimuli^12^ (MOCS) (see Methods)—with measurements recorded over two sessions for both paradigms. We compared sensitivities between stimulus sizes and testing paradigms in addition to evaluating within- and across-session repeatability.

## Methods

### Participants and Procedures

We studied normal-sighted participants across two experimental sessions separated by 2 months ± 2 weeks. Five normal-sighted individuals (two males, three females), 30 to 43 years old (mean ± SD = 34 ± 4.8 years) participated. The research was conducted in compliance with the tenets of the Declaration of Helsinki and was approved by the University of Pennsylvania Institutional Review Board. Before the first experimental session, each participant underwent Early Treatment Diabetic Retinopathy Study visual acuity testing and AOSLO structural imaging to ensure normal vision (best-corrected visual acuity of 20/20 or better), stable fixation, and high-quality AO images of cone photoreceptors within the foveal region. All participants recruited to the study met these criteria. Resolution of the cones within the foveola was not required for inclusion in the study. At the beginning of each imaging session, the subject's preferred eye was dilated with phenylephrine hydrochloride (2.5%) and tropicamide (1%).

### AOSLO Imaging System

We used a custom-built AOSLO to obtain high-resolution images of the fovea, the optical design of which has been described previously.^2^^,^^13^ A summary of the AOSLO imaging system is provided here. An 850-nm super luminescent diode (SLD; Superlum, Cork, Ireland) was used for wavefront sensing, with a custom Shack–Hartmann wavefront sensor and a deformable mirror (DM97-15; ALPAO, Montbonnot-Saint-Martin, France) operating in a closed loop at a frame rate of 8.2 Hz. The power of the 850-nm SLD at the cornea was 10 µW for all sessions. The imaging source was a SLD centered at 795-nm near-infrared wavelength. The average power at the cornea for 795 nm was 57± 2 µW for session 1 and 54 ± 6 µW for session 2. The beams were scanned over a 1° × 1° field of view on the retina, and images of the photoreceptor mosaic were recorded using three photomultiplier tubes (PMTs) arranged in a confocal and non-confocal split detection configuration.^2^^,^^13^^,^^14^ A super-continuum laser generated a visible green light source at 550 nm (Super EXTREME/VARIA, 30-nm full width at half maximum [FWHM] bandwidth; NKT Photonics, Birkerød, Denmark). At the beginning of the session, an image of the fovea was acquired using the 550-nm light to ensure that the 550-nm light was focused on the photoreceptor outer segments. This was done in real time by both maximizing the mean intensity of the visible confocal reflectance signal and viewing the photoreceptor outer segment waveguided image. The power used for imaging with the 550-nm visible light source was 3 µW at the cornea for both sessions, and the exposure time was limited to ensure the cumulative exposure to visible light was lower than 10 times below the maximum permissible exposure set by the American National Standards Institute Safe Use of Lasers.^15^

Participants were dark-adapted for 15 minutes after visible imaging and before initiating psychophysical experiments to allow photopigment regeneration. The average maximum retinal irradiances for the 550-nm light used for the psychophysical stimuli were 43 ± 1 nW/deg^2^ for session 1 and 52 ± 1 nW/deg^2^ for session 2. The background seen by the subjects during the psychophysical experiments was a combination of 550-nm light leak through the acousto-optic modulator (AOM), the infrared imaging light (795 nm), and the wavefront sensing light (850 nm). The cumulative luminance of the background was approximately 28 cd/m^2^. The background appeared reddish to the subjects, indicating that the wavefront and imaging field components of the background dominated the visible light leak component. All irradiances incident on the retina were below the maximum permissible exposure levels as determined by the American National Standards Institute.^15^

### Microperimetry Module

The AO microperimetry instrumentation and software interface used in this study followed previously described methods.^4^^,^^5^ A brief overview of the experimental setup used in the present study is provided here. To perform microperimetry, the AOSLO system incorporated a field-programmable gate array (FPGA)–based image acquisition module combined with a stimulus control module. The confocal PMT signal sent to the native frame grabber of the AOSLO module (HEL 2M QHAL E*; Matrox Electronic Systems, Dorval, QC, Canada) was simultaneously sent to the FPGA module that digitized the PMT signal. Before the start of each imaging session, images of a Ronchi ruling with 0.10° line spacing were acquired using a model eye, and real-time de-warping was implemented to correct for intra-frame distortions caused by the sinusoidal velocity of the resonant optical scanner. An AOM (Brimrose Corporation, Sparks, MD) controlled the signal intensity of the stimulus beam as dictated by the psychophysical algorithms used in each experimental run. Circular stimuli were delivered using a supercontinuum laser (NKT Photonics) centered at 550 nm with a FWHM of 30 nm. A 550-nm light source was chosen, as it excites both long wavelength (L) and medium wavelength (M) cones approximately equally.^16^

**Figure 1.**
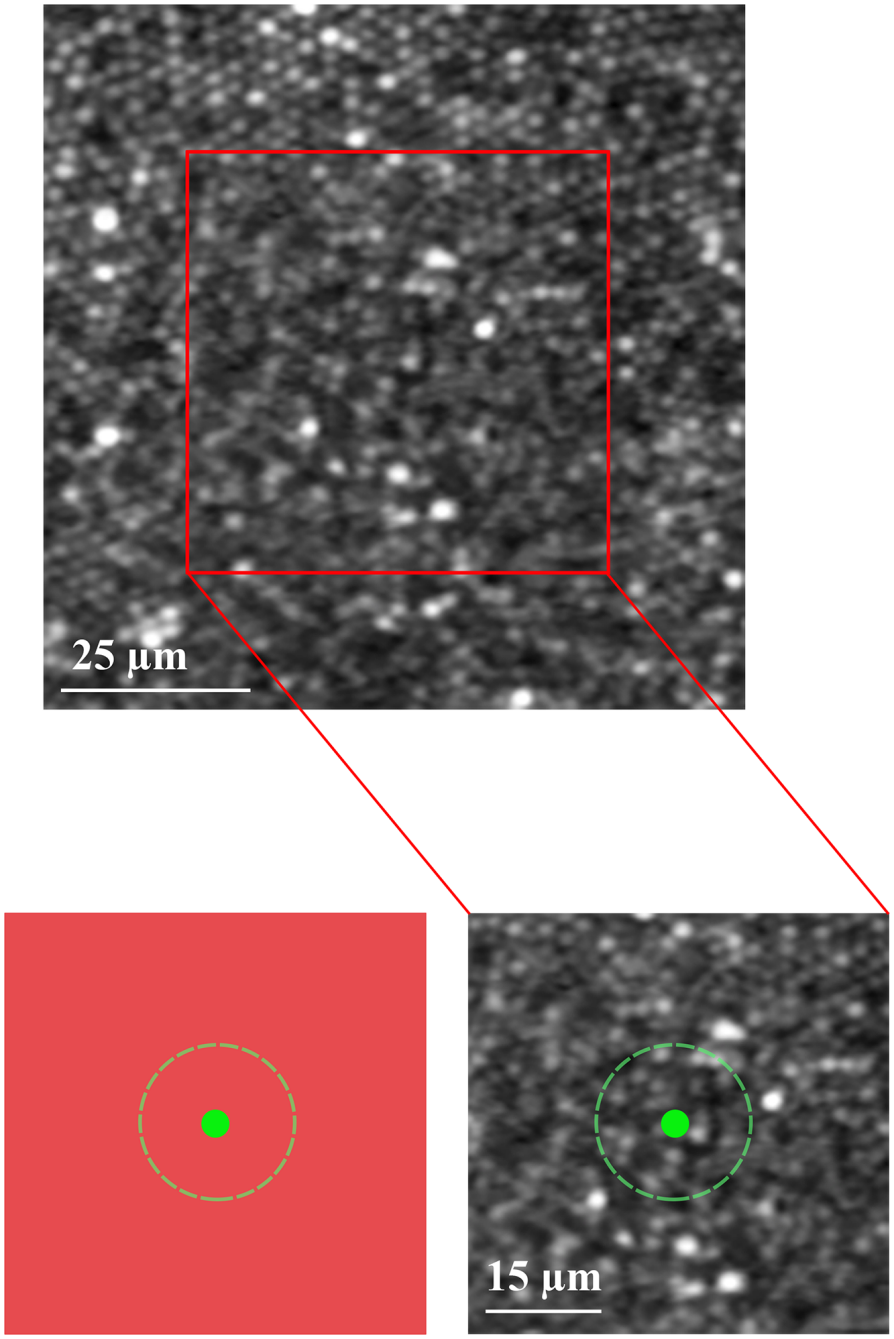
AOSLO image of the cone mosaic in the foveal region of a study participant (*top*). The *bottom left* image shows a schematic of the zoomed central part of the observer's view of a green light stimulus of 1.05 arcmin^2^ (*filled green circle*) and of the area that would be occupied by the 30.4-arcmin^2^ stimulus (*dashed circle*), on a red background. The *bottom right* image shows the zoomed-in portion of the cone mosaic representing the preferred retinal locus within the foveal region with overlayed stimuli. Although they are shown together here, the small and large stimuli were not displayed simultaneously.

Circular stimuli were presented to the center of the 1° × 1° raster scan on every trial (Fig. 1). Subjects were instructed to fixate at the center of the raster, and eye tracking and image stabilization were not employed. In addition, the preferred retinal locus (PRL) of fixation for each subject was imaged while subjects fixated on the center of the imaging square. We did not use an additional fixation target beyond the background supplied by the imaging source, as we did not want to interfere or mask the stimulus presentations, and it is known that individuals can fixate at the center of shapes^17^ and that the preferred retinal locus is stable over days for normal-sighted individuals.^18^

### Psychophysical Testing of Foveal Sensitivity

Circular stimuli (550 nm, Δ30 nm) were presented to the PRL through the AO microperimetry module to measure the foveal sensitivity. Stimuli were presented against the imaging raster, which subtended an approximately 1° × 1° square. The participants pressed a button on a programmable game controller to initiate a trial. The button press triggered the AOSLO to save a 1-second video, during which the stimulus was presented for 187.5 ms (three AOSLO frames). A digital white marker was superimposed on the three stimulus frames in the recorded 1-second AOSLO video to highlight the location where the stimulus was presented. After the stimulus was presented, an audio cue confirmed the stimulus presentation, and participants indicated whether they saw the visual stimulus by pressing the yes/no button. Following this, a second audio cue confirmed that the response had been recorded. The participant could then initiate the subsequent trial. The participant was requested to repeat a trial if the operator identified a blink or eye movement in real time during stimulus presentation. Participants were free to start trials at their own pace. Sensitivities were measured for two stimulus sizes: 1.05 arcmin^2^ (1.16-arcmin diameter) and 30.4 arcmin^2^ (6.2-arcmin diameter). Based on known foveal cone densities,^19^^,^^20^ the 1.06-arcmin^2^ stimulus is expected to be confined to approximately three to five cones, and the 30.4-arcmin^2^ stimulus is expected to span approximately 85 to 130 cones. The larger size approximately matches the smallest stimulus that is routinely delivered in a standard clinical microperimeter or visual field test (Goldmann I stimulus corresponds to a 6-arcmin diameter for comparison^21^). Stimulus levels were converted to decibels (dB) using the following equation:

$$\begin{array}{c} I_{dB}=10*\;\mathrm{lo}g_{10}\left(I/I_{max}\right) \end{array}$$

where *I_max_* represents the maximum stimulus irradiance the system is capable of presenting, and *I* is the stimulus irradiance. Sensitivity (in decibels) was taken as the negative of threshold, with threshold intensity expressed in decibels. The sensitivity ceiling of the AO microperimetry system was 30 dB. As expressed in decibels, stimulus and sensitivity are specified relative to the maximum irradiance in the session and were not corrected for variations in power measurements across sessions.

**Figure 2.**
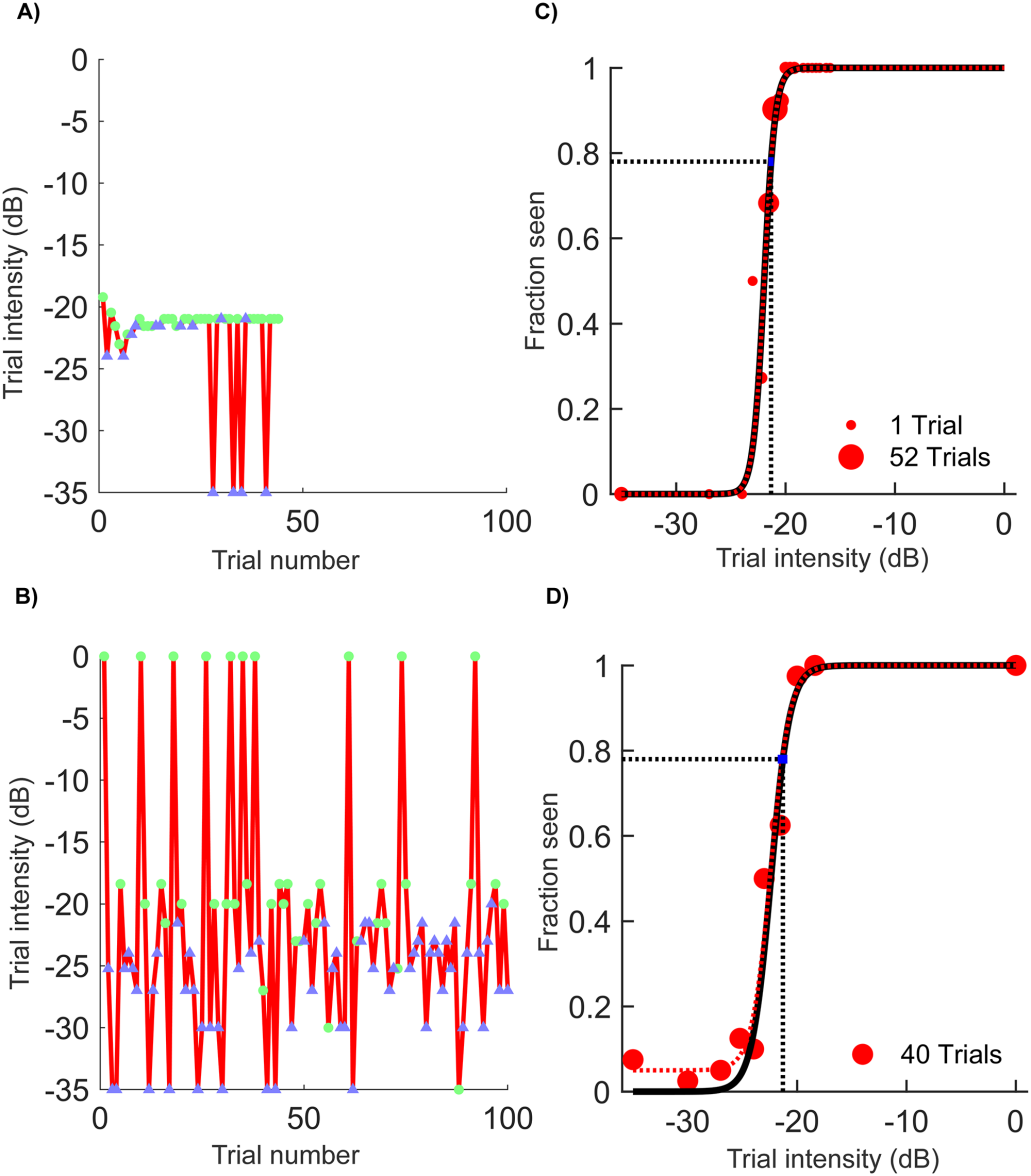
Estimating sensitivity. (**A**) The stimulus presentations for an example QUEST experimental run. (**B**) The stimulus presentations for an example MOCS experimental run for a participant with the 1.05-arcmin^2^ stimulus. (**C, D**) Data from the multiple MOCS and QUEST runs were pooled, and psychometric functions were fit to the MOCS (**C**) and QUEST (**D**) data using the Palamedes Toolbox (*red dashed lines*). The fit psychometric function was then corrected, after setting the false positive and lapse rates to zero (*black lines*). The threshold was chosen as the stimulus intensity that corresponded to the 0.78 fraction seen in the fit psychometric function (*black dashed lines*). The *size* of the data points represents the number of trials tested for a given intensity. The zero-stimulus level was arbitrarily assigned to –35 dB.

We measured sensitivity using adaptive and non-adaptive psychophysical methods, specifically QUEST and MOCS, respectively. QUEST is a Bayesian staircase method that adjusts the stimulus irradiance level for each trial based on the participant's previous responses. QUEST chooses stimulus levels to maximize the expected information gained about threshold on each trial, based on the stimuli and responses on the previous trials.^11^ Each experimental QUEST staircase run consisted of 44 trials, including four catch trials where no stimulus was presented (Fig. 2A).

MOCS is a non-adaptive approach where predetermined stimuli with different irradiance levels are presented.^12^ An initial threshold estimate for each stimulus size was utilized to determine the range of stimulus irradiances presented, designed to ensure that some levels were above and some were below the expected threshold. The initial threshold estimate for each size was chosen based on the outcome of two preliminary 20-trial QUEST staircases, neither of which was included in the final QUEST sensitivity estimation. For each subject and in each session, MOCS experimental runs consisted of nine or 10 different stimulus levels, each repeated 10 times for a total of 90 or 100 trials per run. One stimulus level was at the maximum irradiance presentable through the AOSLO system (on average, 0.047 µW/deg^2^), four were at levels predicted to be above threshold and below the maximum, three or four were predicted to be below threshold, and one was at zero irradiance. The seven or eight stimulus irradiance levels between zero and maximum irradiance were determined using 2-dB spacing around the predicted threshold. Ten unique stimulus levels were presented for all participants for the 1.05-arcmin^2^ size (Fig. 2B). For the 30.4-arcmin^2^ size, however, predicted thresholds were closer to the floor of the AOSLO stimulus modulation range, which resulted in three rather than four stimuli (spaced at 2 dB) below the predicted threshold for four of the total 10 experimental sessions. This resulted in 90 MOCS trials per run for those cases as described in our pre-registration. The other six experimental sessions included 100 MOCS trials per run.

In postprocessing analysis, it was discovered that some of the stimulus intensities thought to be unique for the 30.4-arcmin^2^ condition actually were not. This happened because quantization in the AOM modulation control software caused some nominally distinct stimulus levels to be mapped to the same physical stimulus level. In determining thresholds via the psychometric function fitting (see below), this was corrected for, with physically identical stimuli being pooled for analysis.

The psychophysical testing protocol for a single session proceeded as follows:

1. Training for 30.4-arcmin^2^ stimulus: The threshold for a 30.4-arcmin^2^ circular stimulus was measured three times using an adaptive QUEST staircase containing 20 trials (20 trials × 3 times = 60 trials). The first QUEST staircase was considered training for the participants, and these data were not analyzed. The average threshold from the second and third QUEST staircases was used as the predicted threshold for choosing the stimulus intensities to be tested in the MOCS experimental runs for the 30.4-arcmin^2^ stimulus. The initial stimulus intensity tested for the first trial in the first and second training runs was set to be approximately –25.2 dB. The initial stimulus intensity tested for the first trial in the third training run was set to be approximately –28.2 dB.
2. Training for 1.05-arcmin^2^ stimulus: The threshold for a 1.05-arcmin^2^ circular stimulus was measured three times using an adaptive QUEST staircase with 20 trials (20 trials × 3 times = 60 trials). The first staircase served as a training set for the 1.05-arcmin^2^ stimulus, and these data were not analyzed. The average threshold from the second and third staircases was used as the predicted threshold for the MOCS 1.05-arcmin^2^ stimulus experimental runs. The initial stimulus intensity tested for the first trial in the first and second training runs was set to be approximately –18 dB. The initial stimulus intensity tested for the first trial in the third training run was set to be approximately –21 dB.
3. Experimental run (stimulus size 1): First, the stimulus size to be tested (either 30.4 or 1.05 arcmin^2^) was randomly chosen. Then, sensitivity was measured first using MOCS and second using QUEST. For QUEST, the initial trial was randomly chosen to be either –18 or –21 dB (1.05 arcmin^2^) or –25.2 or –28.2 dB (30.4 arcmin^2^).
4. Experimental run (stimulus size 2): Sensitivity for the second stimulus size (the one not chosen in step 3 above) was then measured using first the MOCS paradigm and then QUEST.
5. Experimental run iteration: Steps 3 and 4 were repeated four times. This resulted in a maximum of 800 trials of MOCS (100 trials per experimental run × 4 runs per stimulus size × 2 stimulus sizes, and 352 trials of QUEST (44 trials per experimental run × 4 runs per stimulus size × 2 stimulus sizes). Thus, a single session consisted of up to 1272 trials (120 trials of QUEST in step 1, and 2800 trials of MOCS and 352 trials of QUEST in steps 3 and 4). The total session time including visible imaging, dark adaptation, training, all experimental trials, and participant-initiated breaks required a time commitment of approximately 90 to 100 minutes.
6. Repeat session: The same psychophysical testing methods were performed in a second session for each participant, conducted 2 months ± 2 weeks after the first session. We used the same starting stimulus levels for all participants as in the first session.

### Psychometric Function Fit and Sensitivity Estimation

The stimulus intensity and response data were pooled across the multiple experimental runs for each stimulus size for each session and testing paradigms (Figs. 2C, 2D). A logistic function implemented in the Palamedes Toolbox^22^ was used to fit the psychometric function for the pooled data, with parameters determined by maximizing the likelihood of the responses. The logistic function can be written as

$$\begin{array}{c} P\left(x\right)=\gamma +\frac{\left(1-\gamma -\lambda \right)}{\left(1+e^{-\beta \left(x-\alpha \right)}\right)} \end{array}$$

where *P*(*x*) is the probability of a seen response at stimulus irradiance *x*, with parameter γ giving the false-positive rate and λ giving the lapse rate. Here, alpha (α) and beta (β) control the location and slope of the psychometric function, respectively, and as such represent the properties of the sensory mechanism.^23^ Gamma (γ) is the false-positive rate parameter that describes how often a seen response was given at zero stimulus intensity. This is sometimes called the guess rate in the literature. Lambda (λ) is the lapse rate and describes how often a not seen response was given at high stimulus intensities. These parameters are included to minimize psychometric function distortion caused by false positives and lapses, but in this study they are not parameters of interest in their own right.^24^ In our fitting, β was constrained to be between 0.5 and 7, γ was constrained to be between 0 and 0.05, and λ was constrained to be between 0 and 0.01; α was not constrained. We constrained these parameters because measured psychometric functions do not always have enough power to lock their values directly from the data, with the specific constraints chosen after preliminary examination of the data. See Kingdom and Prins^25^ for a useful discussion of this issue in the context of the lapse rate; similar considerations apply to the false-positive rate and β, particularly when adaptive methods are used to concentrate trials near the threshold intensity. See Supplementary Table S2 for false-positive and lapse rates estimated directly from the data.

The threshold was taken as the intensity where a logistic function with the fit values of α and β was equal to 0.78, with γ and λ set to 0. This method of correcting for intrusion of false positives and lapses on the estimate of underlying sensitivity is often referred to as “correction for guessing” in the context of high-threshold theory^26^^,^^27^; our method also incorporates lapses.^24^

To determine the confidence interval for a threshold, the data were bootstrapped by resampling with replacement 500 times. Psychometric functions were fit to each run of the bootstrapped data, and thresholds obtained from each of the fits were used to determine the 95% confidence intervals (Fig. 2). The analysis source code is publicly available on GitHub at https://github.com/BrainardLab/AOMicroRepeat.

### Statistical Analyses

Paired two-sample *t*-tests were used for statistical analyses, and Bland–Altman plots^28^ were used to show repeatability. Sensitivity measurements were compared between QUEST and MOCS paradigms using a paired two-sample *t*-test, with *P* < 0.05 considered significant. To assess the agreement between QUEST and MOCS, the limits of agreement (LoA) were calculated as mean ± 1.96 × SD of the differences between the paired measurements. To compare repeatability within and across sessions, data were pooled for the QUEST and MOCS paradigms. For within-session comparisons, session data were split into two groups randomly by experimental run, psychometric functions were fit to the split data as described above. The coefficient of repeatability was calculated as 1.96 × SD of the differences between paired measurements both within and between sessions for each stimulus size. The significance of within and between session differences was evaluated using a paired two-sample *t*-test, considering *P* < 0.05 as significant.

### Preregistration of the Study

The study design was preregistered with the Open Science Framework prior to beginning all data collection and associated analysis (https://osf.io/bzf6g/overview). Deviations from our preregistered design can be found in Supplementary Table S1.

## Results

### Comparison of Sensitivities Measured With MOCS and QUEST

Sensitivity was measurable for both stimulus sizes and both testing paradigms in all five participants in both experimental sessions. In all cases, the adaptive QUEST procedure converged and yielded data similar to those shown in Figure 2A. For the smaller stimulus (1.05 arcmin^2^), the mean ± standard error of the mean (SEM) sensitivity values for MOCS and QUEST, respectively, were 20.6 ± 0.3 dB and 20.3 ± 0.5 dB in session 1 and 21.2 ± 0.3 dB and 21.5 ± 0.1 dB in session 2. For the larger stimulus (30.4 arcmin^2^), sensitivity was measured as 28.4 ± 0.5 dB (MOCS) and 28.2 ± 0.5 dB (QUEST) in session 1 and 27.9 ± 0.4 dB (MOCS) and 28.5 ± 0.4 dB (QUEST) in session 2. As expected, sensitivity was higher for the larger stimulus (paired sample *t*-test, *P* < 0.001 for both session 1 and session 2 and for both QUEST and MOCS paradigms). The total false-positive rates in session 1 were 2.5% and 1.1% for the small and large stimuli, respectively, using the MOCS testing paradigm, and 1.3% and 0% for the small and large stimuli using the QUEST testing paradigm. In session 2, total false-positive rates for the small and large stimuli were 2.2% and 1.6%, respectively, using the MOCS paradigm, and the QUEST paradigm resulted in a false-positive rate of 1.3% for both stimulus sizes. (Supplementary Table S2).

**Figure 3.**
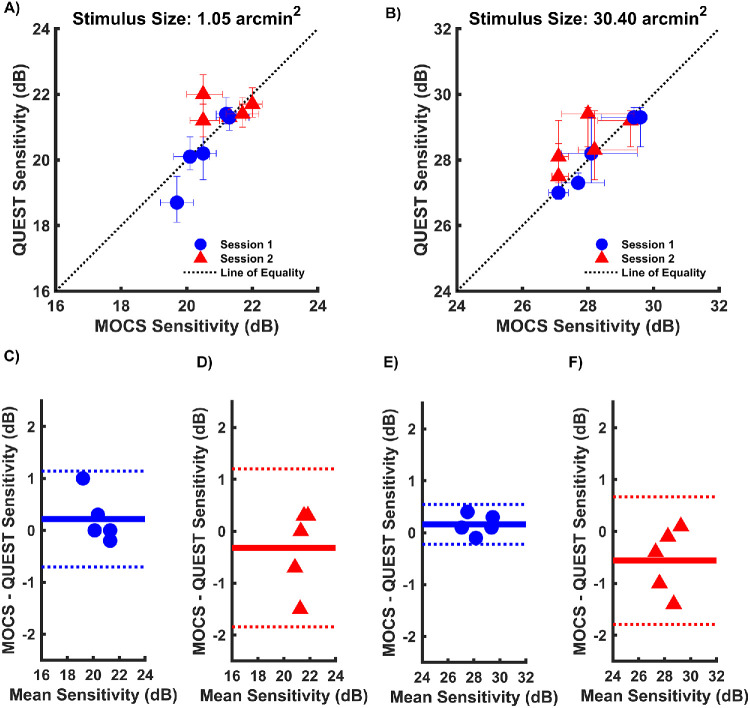
(**A, B**) These plots illustrate the agreement between sensitivity measurements acquired from MOCS and QUEST procedures for the small and large stimulus sizes, respectively. The *dotted black line* represents the line of equality. *Blue circles* and *red triangles* represent session 1 and session 2 data, respectively. *Horizontal* and *vertical error bars* on the markers represent the 95% confidence intervals obtained from bootstrapping. (**C–F**) Bland–Altman plots for measurements made using QUEST and MOCS paradigms. The *x*-axis represents the mean of paired MOCS and QUEST sensitivity measurements, and the *y*-axis represents the difference between the paired sensitivity measurements (MOCS minus QUEST). *Thick lines* represent the mean difference between MOCS and QUEST, and the *dotted lines* represent the 95% LoA that are ±1.96 SD from the mean. Shown are the session 1 and 2 data, respectively, for the small stimulus size (**C, D**) and the session 1 and 2 data, respectively, for the large stimulus size (**E, F**).

Measurements for individual study participants show that the QUEST and MOCS sensitivities were similar (Fig. 3A). Bland–Altman plots revealed that the LoA for the difference in the sensitivity measurements between MOCS and QUEST included zero for both stimulus sizes in both experimental sessions (Figs. 3B–E). A paired two-sample *t*-test revealed that the measured sensitivities were not significantly different when measured using the QUEST or MOCS paradigms (for the smaller stimulus size, *P* = 0.36 for session 1 and *P* = 0.41 for session 2; for the larger stimulus size, *P* = 0.14 for session 1 and *P* = 0.12 for session 2). Because there were no statistical differences between the MOCS and QUEST measurements for either stimulus size in either session, all further analyses pooled trials collected using MOCS and QUEST paradigms into one dataset for each stimulus size for each session.

### Within-Session Repeatability

**Figure 4.**
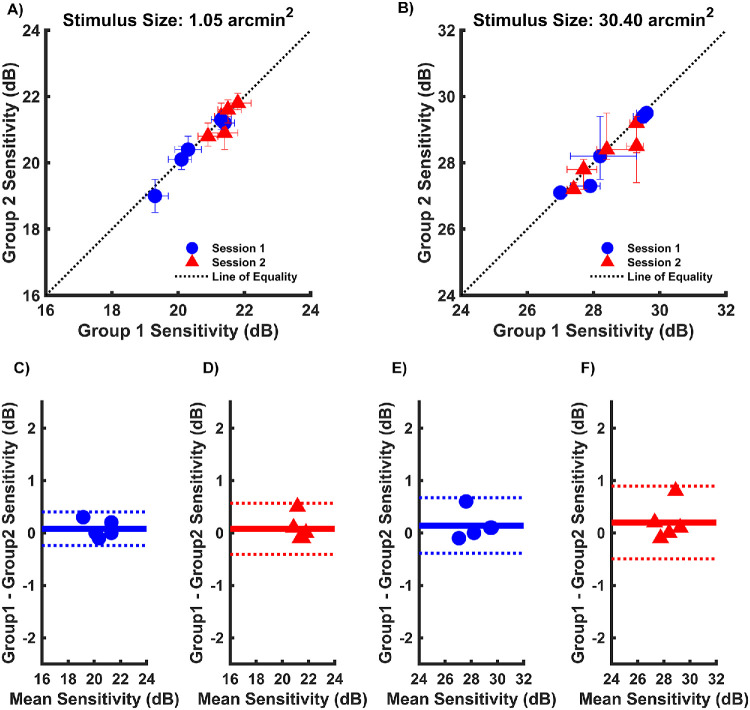
(**A, B**) These plots illustrate the agreement between sensitivity measurements within-session performed by splitting data within each session into two halves. The *x*-axis (group 1) represents one-half of the data, and the *y*-axis (group 2) represents the other half of the data. The *dotted black line* represents the line of equality. *Blue circles* and *red triangles* represent session 1 and session 2 data, respectively. *Horizontal* and *vertical error bars* on the markers represent the 95% confidence intervals obtained from bootstrapping. (**C–F**) The panels show the Bland–Altman plots for within-session sensitivity measurements. *Thick lines* represent the mean difference, and the *dotted lines* represent the 95% LoA that are ±1.96 SD from the mean. Shown are the session 1 and 2 data, respectively, for the small stimulus size (**C, D**) and the session 1 and 2 data, respectively, for the large stimulus size (**E, F**).

For within-session repeatability, we pooled the data from the four QUEST experiments (44 × 4 trials) and four MOCS experiments (100 × 4 trials) for each session and stimulus size. The four experimental runs were then randomly divided into two groups of two experimental runs, and sensitivity measurements for each group were compared. Figure 4A shows good agreement in sensitivity between the two groups. Again, the LoA in the Bland–Altman analysis (Figs. 4B–E) encompassed zero, suggesting no difference between the sensitivities of the two groups. For session 1, the paired-sample *t*-test did not show a significant difference between groups for both 1.05 arcmin^2^ (20.5 ± 0.4 dB and 20.4 ± 0.4 dB; *P* = 0.34) and 30.4 arcmin^2^ (28.4 ± 0.5 dB and 28.3 ± 0.5 dB; *P* = 0.31) stimulus sizes. Similarly, for session 2, there was no significant difference between groups for 1.05 arcmin^2^ (21.4 ± 0.1 dB and 21.3 ± 0.2 dB; *P* = 0.51) or 30.4 arcmin^2^ (28.4 ± 0.4 dB and 28.2 ± 0.3 dB; *P* = 0.27). The coefficient of repeatability for the 1.05-arcmin^2^ stimulus was 0.3 dB for session 1 and 0.5 dB for session 2. For the 30.4-arcmin^2^ stimulus, the coefficients of repeatability were 0.5 dB and 0.7 dB for session 1 and session 2, respectively.

### Intersession Repeatability

**Figure 5.**
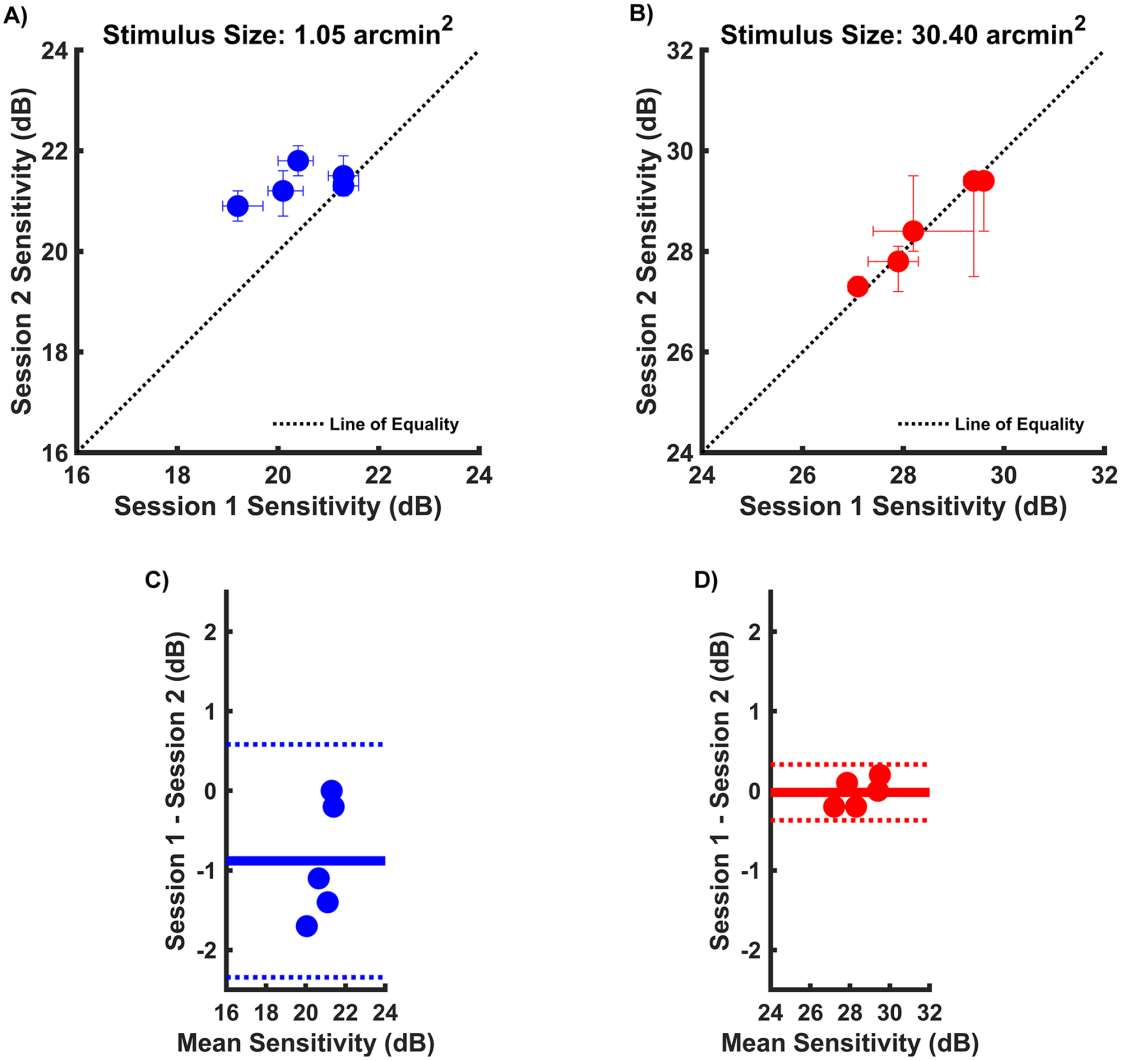
Analysis of between-session repeatability. (**A, B**) These plots illustrate the similarity between session 1 and session 2 sensitivity measurements for the small and large stimulus sizes, respectively. The *dotted black line* represents the line of equality. *Blue* and *red circles* represent the sensitivity measurements obtained from a smaller and larger stimulus size, respectively. *Horizontal* and *vertical error bars* represent the 95% confidence intervals obtained from bootstrapping. (**C, D**) Bland–Altman plots for comparing sensitivity measurements between sessions, where *blue* represents the 1.05-arcmin^2^ stimulus; *red*, the 30.4-arcmin^2^ stimulus. *Thick lines* represent the mean difference between groups, and the *dotted lines* represent the 95% LoA that are ±1.96 SD from the mean.

Sensitivity measurements acquired in sessions 1 and 2 showed close agreement across sessions for both stimulus sizes (Fig. 5A). Again, the Bland–Altman analysis showed that the LoA for the difference in measurements included zero (Figs. 5B, 5C). A paired-sample *t*-test did not reveal a significant difference (*P* = 0.06) in sensitivity between session 1 (20.5 ± 0.4 dB) and session 2 (21.3 ± 0.2 dB) for the 1.05-arcmin^2^ condition. Similarly, sensitivity measurements were not significantly different (*P* = 0.81) between session 1 (28.4 ± 0.5 dB) and session 2 (28.5 ± 0.4 dB) for the 30.4-arcmin^2^ condition. The coefficient of repeatability was 1.5 dB for the 1.05-arcmin^2^ stimulus and 0.4 dB for the 30.4-arcmin^2^ stimulus.

## Discussion

Our findings demonstrate that retinal sensitivity can be reliably obtained at the PRL using AO microperimetry. Sensitivity was lower for the small stimulus in comparison to the large stimulus, and sensitivities obtained using the MOCS and QUEST testing paradigms were not significantly different. In addition, foveal sensitivities were repeatable both within and across sessions for the normal-sighted participants included in this study.

Clinical microperimeters, such as the MP-3 (Nidek, Aichi, Japan) and the MAIA (iCare Finland Oy, Vantaa, Finland), test sensitivity at dozens of locations across the macula and have demonstrated adequate repeatability for clinical use in both healthy and diseased cohorts. Commonly used metrics in clinical microperimetry include pointwise sensitivity, which is the sensitivity measured at one tested retinal location, and mean sensitivity, which is the mean of the sensitivities measured from all tested retinal locations. Palkovits et al.^29^ found the coefficient of repeatability for the Nidek MP-3 to be ±3.3 dB in healthy and ±5 dB in diseased patients for pointwise sensitivity measurements. In addition, they showed that mean sensitivity was less variable than pointwise sensitivity with mean sensitivity coefficients of repeatability of ±1.2 dB in controls and ±1.6 dB for macular diseased patients.^29^ Coulibaly et al.^30^ found that the mean coefficient of repeatability for the MAIA microperimeter was ±4.61 dB in healthy aged subjects, and Wu et al.^31^ found a pointwise coefficient of repeatability of 3.81 dB in normal-sighted individuals. Our AO microperimetry coefficients of repeatability were 0.4 dB for the larger stimulus and 1.5 dB for the smaller stimulus. There are several potential causes for the lower variability for the AO microperimetry found in the present study in comparison to the studies using clinical devices. First, our protocol only tested one retinal location, the fovea, so any potential uncertainty caused by the location of stimulus presentation was eliminated. In addition, participants had significantly more trials at this one location in comparison to typical testing procedures for clinical microperimetry devices.

Assessing repeatability within and across sessions is important for the interpretation of studies that follow sensitivity measurements longitudinally. We found that foveal sensitivities were repeatable both within and across sessions. This shows that the limited training performed at the start of the experiment was sufficient for conducting the experiment. Importantly, sensitivity did not significantly increase in the second session, so we concluded that the subjects’ experiences from the first session did not alter measurements made in the second session. Finally, the variability observed between sessions for an individual participant was small compared to the variations observed between participants. Thus, we conclude that a single measurement of sensitivity is sufficiently accurate for determining differences in sensitivities between individuals.

The repeatability for two different sized stimuli was tested in the current study. The smaller stimulus was chosen as it was expected that the threshold for this stimulus size would fall close to the center of the AO microperimetry dynamic range for presentable contrasts, and indeed this was the case. The main advantage of AO microperimetry is the ability to test retinal function at high spatial resolution, but this requires the presentation of small stimuli.^8^^–^^10^ Some patients however, will present with reduced acuities and thus may not be able to see small stimuli regardless of their intensity. Thus, we also decided to test a larger stimulus size as it was considered important to determine repeatability in control participants using stimuli that are likely to be needed in future applications of the technique to visually impaired patients. Previous studies have reported that the variability in sensitivity is smaller for larger stimuli.^32^^,^^33^ This is in agreement with the current study where we found the coefficient of repeatability to be higher for the smaller stimuli tested (1.5 dB for 1.05 arcmin^2^ vs. 0.4 dB for 30.4 arcmin^2^). We also found that the smaller stimulus (1.06 arcmin^2^) yielded a higher overall false-positive rate (2.3%) compared to the larger stimulus (30.4 arcmin^2^, 1.3%). This is also in agreement with observers having more certainty for the larger stimulus.

Sensitivities for the 30.4-arcmin^2^ stimulus were on average 7.55 dB higher than those for the 1.06-arcmin^2^ stimulus. To relate our findings to established physiological models, we compared the sensitivity difference between stimulus sizes to those measured in Tuten et al.^5^ The range of sensitivities measured in the current study were close to the range of sensitivities measured in Tuten et al.^5^ for both the 1.06-arcmin^2^ and the 30.4-arcmin^2^ stimulus sizes after converting between the different units reported in the studies. Although the 1.06-arcmin^2^ stimulus operates within the foveal Ricco's area (2.4 arcmin diameter), the 30.4-arcmin^2^ stimulus exceeds this critical size. Our observed sensitivity difference between the two stimulus sizes is in agreement with findings considering the neural pooling known to occur over different stimulus sizes.^5^

Previous studies have also shown that the variability in sensitivities measured using clinical automated perimetry in normal subjects doubles when moving from the central 10° to the peripheral 20° to 30°.^34^^,^^35^ Indeed, studies have shown that the within-session, between-session, and inter-subject variability was significantly lower in the central 10° visual field (±1–2 dB) compared to the peripheral 30° visual field (±3–6 dB) for healthy controls.^33^^,^^35^^,^^36^ Variability in sensitivity also has been shown to increase within regions of sensitivity loss in patients with glaucoma,^33^^,^^36^ age-related macular degeneration,^37^ and several inherited retinal degenerations.^31^^,^^38^^–^^40^ Future studies using AO microperimetry could be undertaken to determine the coefficient of repeatability for sensitivity measurements at retinal eccentricities outside of the fovea and for patients with visual impairments.

Another factor to consider when assessing variability is the retinal irradiance for the light sources. We measured power at the cornea for all light sources in both sessions for all participants, but we did not precisely match powers across the different sessions or across study participants. The mean irradiance levels for the green stimulus (550 nm, Δ30 nm) were consistently higher in session 2 compared to session 1. In addition, the irradiance of the 790-nm imaging source varied up to 6.4% between sessions and participants. Variabilities in irradiance for both the stimulus light and the imaging source affect stimulus contrast at the retina, which in turn could lead to increased variability across sessions and between participants. Indeed, variability in the irradiances across sessions could be a reason for the borderline statistical finding (*P* = 0.06) for the smaller stimulus. Future experiments could consider using calibrated light levels for all sources across all sessions in a study. We note that, even without this correction, sensitivity measurements were highly repeatable, showing that day-to-day variations in irradiance equal to or less than the present study yield an acceptable measure of sensitivity repeatability.

To our knowledge, all clinical devices measuring retinal sensitivities use adaptive procedures as opposed to constant stimuli in order to identify sensitivity expeditiously. The present study tested one adaptive procedure, QUEST, in comparison with MOCS and showed that the adaptive procedure arrived at the same sensitivity. Our QUEST procedure used 56% fewer trials than MOCS, which resulted in an experimental advantage requiring 56% less time. Although we did not assess the variability of QUEST and MOCS measurements separately, Figure 3A shows that the bootstrapped error bars for individual measurements are similar between the two methods. We thus recommend that future experiments utilize the QUEST experimental design, perhaps with the addition of a few trials at high irradiance to anchor the high portion of the psychometric function and allow for an estimation of lapse rate.

## Conclusions

The present study demonstrated the repeatability of within-session and between-session measurements of foveal sensitivity as measured with AO microperimetry. The study also shed light on considerations for efficient and accurate experimental designs that can be used for translating AO microperimetry measurements to study retinal disease and its progression.

## Supplementary Material

Supplement 1

Supplement 2
