# Supplementary material for: Repeatability of Foveal Sensitivity Measured Using Adaptive Optics Microperimetry
Source: Transl Vis Sci Technol. 2026 Aug 5;15(8):3. doi: 10.1167/tvst.15.8.3 (PMC13450651; doi:10.1167/tvst.15.8.3)
Supplement: Supplementary file 1 [file tvst-15-8-3_s001.pdf]

Supplemental Table 1: List of deviations from preregistration document

Statement (summarized) from Preregistration	Deviation and Rationale
Examine thresholds using a subset of the data where trials with blinks, changes in image quality, or high eye movements are discarded	We found thresholds were repeatable including all trials in the analysis and decided it was unnecessary to examine these subsets of trials.
Examine thresholds using only 90% of the trials with the lowest eye motion	We found thresholds were repeatable including all trials in the analysis and decided it was unnecessary to examine this subset of trials.
Examine thresholds for trials exhibiting less than 2.5 arcmin of movement during stimulus delivery	We found thresholds were repeatable including all trials in the analysis and decided it was unnecessary to examine this subset of trials.
Registration and plot of stim locations	We did not plot stimulus locations combined across trials because we decided it was unnecessary to examine subsets of trials based on eye motion as thresholds were repeatable using all trials.
To account for the different number of trials acquired for MOCS and QUEST, we will analyze thresholds for MOCS using the first 160 trials that are evenly distributed across the tested stimulus intensities, in addition to analyzing the threshold for MOCS using all trials.	We decided the analysis with only a subset of the MOCS trials was unnecessary, and instead include in the discussion the idea that MOCS and QUEST yielded the same threshold but QUEST did so with fewer trials.
Thresholds for QUEST and MOCS will be compared again using a paired-samples Wilcoxon test.	We report the results using a paired t-test as it is a more sensitive test. Results from paired Wilcoxon tests showed thresholds were also not significantly different.
Intrasession and intersession comparisons done for each condition (MOCS and QUEST)	Because thresholds for MOCS and QUEST were not statistically different from each other, we decided to combine all trials from both methods for intra- and intersession comparisons. We did not analyze intrasession and intersession repeatability for MOCS and QUEST separately.
Measure any differences in stimulus locations between sessions in post-analysis	We did not calculate differences in stimulus locations between sessions as we decided this was unnecessary because thresholds were repeatable between sessions using the participant preferred retinal locus at each session.
Data collection protocol for identifying MOCS stimuli	For one subject, the MOCS estimated threshold was typed incorrectly into the stimuli presentation code, resulting in slightly shifted stimuli intensities for 3 of the 4 MOCS experimental runs. The stimuli still straddled the threshold, so we decided to include those data and estimate thresholds using the trials captured in sequence as described by the experimental code. Thus, for this subject there were more

	unique stimuli represented in the MOCS data, but the total number of trials remained the same as for other subjects.
We may also assess between and within session threshold measurements for QUEST and MOCS using Spearman's rank correlation, Bland and Altman plots, and a paired-samples Wilcoxon test.	We provide Bland-Altman plots and report paired t-tests and the coefficient of repeatability, we do not report Spearman's rank correlation or Wilcoxon tests.
Visible light image will be used for determining the focus and stimulus offset caused by transverse chromatic aberration.	We used the visible light image to determine the stimulus focus on the photoreceptors but did not calculate the offset caused by transverse chromatic aberration as we use the preferred retinal locus of fixation and did not track eye motion.
Correction for guessing done using $$p_{corr} = \frac{p_{hit} - p_{fa}}{1 - p_{fa}}$$	Correction for guessing done differently as described in the methods.
