# Supplementary material for: Repeatability of Foveal Sensitivity Measured Using Adaptive Optics Microperimetry
Source: Transl Vis Sci Technol. 2026 Aug 5;15(8):3. doi: 10.1167/tvst.15.8.3 (PMC13450651; doi:10.1167/tvst.15.8.3)
Supplement: Supplementary file 2 [file tvst-15-8-3_s002.pdf]

**Table 2: Number of false positive and false negative trials per psychophysical testing method, session, and study participant.** False positive rates are provided as the number of times a participant responded seeing the stimulus on trials when no stimulus was presented (numerator), relative the number of trials where no stimulus was presented (denominator). Lapse rates are provided as the number of times a participant responded not seeing the stimulus on trials where a stimulus at least 6 dB above the estimated threshold was presented. There are not enough QUEST trials at least 6 dB above threshold to make estimating the lapse rates meaningful, so these are not provided for the QUEST data.

A) **False Positive Rates, Session 1**

| Subject ID | MOCS (1.05 arcmin$^2$) | QUEST (1.05 arcmin$^2$) | MOCS (30.4 arcmin$^2$) | QUEST (30.4 arcmin$^2$) | Total | Percent |
|---|---|---|---|---|---|---|
| 11002 | 0/40 | 0/16 | 2/160 | 0/16 | **2/232** | **0.9%** |
| 11108 | 0/40 | 0/16 | 0/160 | 0/16 | **0/232** | **0%** |
| 11118 | 4/40 | 1/16 | 3/160 | 0/16 | **8/232** | **3.4%** |
| 11119 | 1/40 | 0/16 | 5/200 | 0/16 | **6/272** | **2.2%** |
| 11125 | 0/40 | 0/16 | 0/200 | 0/16 | **0/272** | **0%** |
| Total (n=5) | **5/200 (2.5%)** | **1/80 (1.3%)** | **10/880 (1.1%)** | **0/80 (0%)** | **16/1240** | **1.3%** |

B) **False Positive Rates, Session 2**

| Subject ID | MOCS (1.05 arcmin$^2$) | QUEST (1.05 arcmin$^2$) | MOCS (30.4 arcmin$^2$) | QUEST (30.4 arcmin$^2$) | Total | Percent |
|---|---|---|---|---|---|---|
| 11002 | 3/40 | 0/16 | 3/160 | 0/16 | **6/232** | **2.6%** |
| 11108 | 1/70 | 0/16 | 0/200 | 0/16 | **1/302** | **0.3%** |
| 11118 | 1/40 | 0/16 | 1/200 | 0/16 | **2/272** | **0.7%** |
| 11119 | 0/40 | 1/16 | 11/160 | 1/16 | **13/232** | **5.6%** |
| 11125 | 0/40 | 0/16 | 0/200 | 0/16 | **0/272** | **0%** |
| Total (n=5) | **5/230 (2.2%)** | **1/80 (1.3%)** | **15/920 (1.6%)** | **1/80 (1.3%)** | **22/1310** | **1.7%** |

**C) Lapse Rates, Session 1**

| Subject ID | MOCS (1.05 $arcmin^2$) | MOCS (30.4 $arcmin^2$) | Total | Percent |
|---|---|---|---|---|
| 11002 | 0/40 | 0/40 | **0/80** | **0%** |
| 11108 | 0/40 | 1/40 | **1/80** | **1.2%** |
| 11118 | 0/40 | 0/40 | **0/80** | **0%** |
| 11119 | 1/40 | 0/40 | **1/80** | **1.2%** |
| 11125 | 0/40 | 0/40 | **0/80** | **0%** |
| Total (n=5) | **1/200 (0.5%)** | **1/200 (0.5%)** | **2/400** | **0.5%** |

**D) Lapse Rates, Session 2**

| Subject ID | MOCS (1.05 $arcmin^2$) | MOCS (30.4 $arcmin^2$) | Total | Percent |
|---|---|---|---|---|
| 11002 | 0/40 | 0/40 | **0/80** | **0%** |
| 11108 | 0/40 | 0/40 | **0/80** | **0%** |
| 11118 | 0/40 | 0/40 | **0/80** | **0%** |
| 11119 | 0/40 | 0/40 | **0/80** | **0%** |
| 11125 | 0/40 | 0/40 | **0/80** | **0%** |
| Total (n=5) | **0/200 (0%)** | **0/200 (0%)** | **0/400** | **0%** |
